# Isolated Ascending Aortic Aneurysms: Five-Year Comparative Outcomes between Modified Wrapping Technique and Supracoronary Tube Replacement

**DOI:** 10.5761/atcs.oa.26-00030

**Published:** 2026-06-16

**Authors:** Henri Dubrulle, Augustin Coisne, Samy Aghezzaf, Adham Bardeesi, Antoine Bical, François Pontana, David Montaigne, Thomas Modine, André Vincentelli, Mohammad Koussa

**Affiliations:** 1 Department of Cardiology and Thoracic Surgery, Orleans University Hospital, Orleans, France; 2 Department of U1011-EGID, Inserm, The University of Lille, Lille, France; 3 Department of Cardiovascular Surgery, Lille University Hospital, Lille, France; 4 Cardiovascular Research Foundation, New York City, NY, USA; 5 UMCV, Haut-Lévêque Cardiology Hospital, Bordeaux University Hospital (CHU de Bordeaux), Pessac, France

**Keywords:** aortic aneurysm, wrapping technique, supracoronary tube, arterial stiffness, aortic surgery

## Abstract

**Purpose:**

This study aimed to compare outcomes of the modified wrapping technique (MWT) and supracoronary tube replacement (STR) for isolated ascending aortic aneurysms, focusing on perioperative safety, 5-year survival, and arterial stiffness.

**Methods:**

This retrospective, single-center study included 205 patients undergoing MWT (n = 91) or STR (n = 114) between 2006 and 2019 for isolated ascending aortic aneurysms ≥50 mm or rapidly progressing lesions. A prospective substudy evaluated arterial stiffness using the SphygmoCor system in a matched subgroup of 40 patients. The primary endpoint was 5-year survival; secondary endpoints included perioperative outcomes, prosthetic infection, and hemodynamic indices.

**Results:**

MWT showed significantly shorter cardiopulmonary bypass and aortic cross-clamp times (p <0.0001) and reduced hospital stay (10.5 ± 0.3 vs. 13.5 ± 0.8 days; p = 0.001). In-hospital mortality tended to be lower with MWT (0% vs. 4.4%; p = 0.06). Five-year survival was comparable between groups (88.1% vs. 83.4%; p = 0.17). Prosthetic infection was significantly lower in the MWT group (1.1% vs. 7.0%; p = 0.04). Hemodynamic assessment demonstrated lower pulse wave velocity and aortic augmentation index in the MWT group.

**Conclusions:**

MWT was associated with shorter operative times and lower prosthetic infection rates while maintaining similar early and mid-term survival compared with STR. These findings suggest MWT as a valuable alternative in selected patients.

## Abbreviations

AA
: ascending aorta
AIx
: aortic augmentation index
BMI
: body mass index
CKD
: chronic kidney disease
CPB
: cardiopulmonary bypass
COPD
: chronic obstructive pulmonary disease
CT
: computed tomography
DPTI
: diastolic pressure–time integral
ECMO
: extracorporeal membrane oxygenation
GFR
: glomerular filtration rate
LVEF
: left ventricular ejection fraction
ICU
: intensive care unit
MWT
: modified wrapping technique
PP
: pulse pressure
PWV
: pulse wave velocity
RM
: reflection magnitude
SPTI
: systolic pressure–time integral
STR
: supracoronary tube replacement

## Introduction

The management of ascending aortic aneurysms is well established and typically involves complete replacement of the ascending aorta (AA).^1,2)^ Although this surgical approach provides durable long-term results,^3,4)^ it is associated with well-documented perioperative risks.^3–5)^ In light of updated clinical practice guidelines,^1)^ there is growing interest in earlier surgical strategies aimed at reducing operative morbidity, particularly through shorter operative times.

External aortic wrapping has emerged as one such technique, demonstrating encouraging early outcomes.^6–8)^ Nevertheless, concerns persist regarding its long-term safety, particularly with respect to aneurysm recurrence, aortic dissection, prosthetic migration, and aortic wall erosion.^9–15)^

To address these challenges, a modified wrapping technique (MWT) was developed at Lille University Hospital. We hypothesized that this approach would reduce complication rates and improve hemodynamic outcomes by preserving the native aortic wall.

The primary aim of this study was to evaluate the safety and efficacy of the MWT in comparison with supracoronary tube replacement (STR) of the AA by analyzing the 5-year outcomes of a large patient cohort. A secondary objective was to compare aortic stiffness parameters associated with each technique within a comparable subgroup derived from this cohort.

## Materials and Methods

### Study design

This retrospective, single-center study was conducted at Lille University Hospital, France.

### Study population

All consecutive patients who underwent an MWT or STR for isolated ascending aortic aneurysm between January 2006 and December 2019 were included. Follow-up was obtained through systematic review of hospital medical records and, when necessary, direct telephone contact with patients. If patients could not be reached, their general practitioner or referring cardiologist was contacted to ascertain vital status, last clinical information, and most recent imaging follow-up.

### Inclusion and exclusion criteria

Inclusion criteria were a tubular AA or sinotubular junction diameter ≥50 mm, or rapid aneurysm progression >4 mm/year on computed tomography (CT), in accordance with the clinical practice guidelines in effect at the time of surgery.^2)^

Patients undergoing emergency procedures, those with suspected genetic connective tissue disorders, those requiring additional cardiac procedures (such as aortic valve replacement or coronary artery bypass grafting), aortic arch procedures (including hemi-arch replacement), or those with a history of prior cardiac or aortic surgery were excluded to ensure a homogeneous study population and to minimize confounding related to redo procedures.

The choice between the MWT and STR was not randomized and reflected real-world surgical practice during the study period, incorporating the operating surgeon’s experience, patient age, anticipated operative risk, and institutional practice.

### Ethics

All patients provided written informed consent and were enrolled in the POMI-AF cohort (ClinicalTrials.gov: NCT03376165; ID-RCB: 2017-A00852-51). The study was approved by the institutional review board and conducted in accordance with ethical standards.

### Surgical techniques

#### MWT (Fig. 1)

**Fig. 1:**
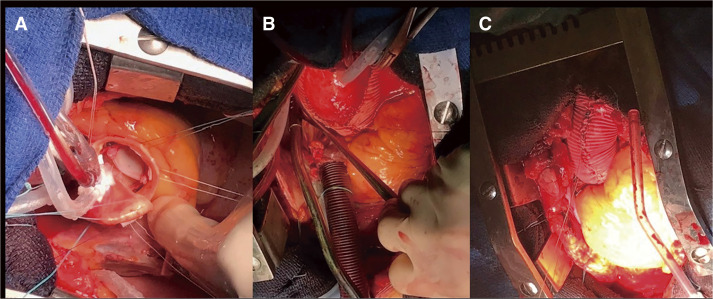
Perioperative aspects of the modified wrapping procedure. (**A**) U-stitches ensuring proximal fixation. (**B**) Proximal fixation with the overlock suture passed. (**C**) Final appearance of the modified wrapping technique.

Unlike the conventional wrapping approach, the MWT requires cardiopulmonary bypass (CPB) with aortic cross-clamping. Arterial cannulation is performed centrally on the distal AA, adjacent to the brachiocephalic trunk, in a macroscopically healthy segment. Venous cannulation is conventionally achieved through the right atrium.

The modified wrapping procedure begins with aortic cross-clamping and administration of antegrade cardioplegia through the aortic root. A limited aortotomy of approximately 2 cm is then performed to allow passage of pledgeted 2-0 polyester U-stitches through the aortic wall, ensuring secure proximal graft anchoring: a total of 6 sutures are placed, including 3 at the level of the commissures and 3 at the level of the sinuses. The aortotomy is subsequently closed, with de-airing of the left cardiac chambers, followed by aortic unclamping.

The vascular graft, previously opened longitudinally, is then passed posteriorly around the AA while carefully avoiding posterior folds. The proximal portion of the graft is secured using the previously placed U-stitches. CPB weaning is initiated during the early phase of the edge-to-edge graft suture, allowing gradual repressurization of the aorta and ensuring optimal graft adaptation. Once weaning from CPB is completed, all cannulas are removed. The distal graft suture is then completed up to the level of the brachiocephalic trunk, allowing complete coverage of the previously cannulated AA and cardioplegia entry sites, providing an additional hemostatic effect and potentially reducing the risk of pseudoaneurysm formation.

This MWT allows optimal graft positioning during aortic pressurization while limiting the risk of graft plication. It also provides improved hemostasis.

### STR

STR was performed using standard surgical techniques under CPB and aortic cross-clamping, with the same standard central cannulation technique.

The same type of polyester vascular graft was used in both groups. Graft sizing differed according to the surgical technique: in the MWT group, graft diameter (34 or 36 mm) was selected based on the external aortic diameter, whereas in the STR group, graft sizes typically ranged from 28 to 32 mm and were based on the internal aortic diameter.

In both MWT and STR techniques, particular attention was paid to selecting the aortic cross-clamping site, which was applied to macroscopically healthy segments, avoiding calcified or fragile areas. Cerebral perfusion was continuously monitored intraoperatively using near-infrared spectroscopy.

### Clinical outcomes

For the main cohort, the primary endpoint was 5-year survival. Secondary endpoints included perioperative safety (e.g., reoperation for bleeding and early complications) and efficacy outcomes, including late dilatation, dissection, endocarditis, prosthetic infection, and prosthetic migration. Endocarditis and prosthetic infection were diagnosed according to the modified Duke criteria^16)^ and confirmed by a multidisciplinary heart team.

All patients underwent at least 2 CT scans during follow-up, including 1 performed at or beyond 5 years. CT imaging was used to assess aortic diameter evolution, graft position, and the occurrence of late complications such as redilatation, aortic wall thinning, or dissection.

### Prospective exploratory hemodynamic substudy

A prospective exploratory substudy was conducted between 2017 and 2019 and included 40 consenting patients who had undergone surgery between 6 months and 2 years prior to enrollment (20 per group: MWT and STR). Patients were selected to achieve comparable baseline characteristics between groups; this substudy was exploratory and was not intended to provide a statistically representative sample of the full cohort.

The objective of this substudy was to compare ascending aortic hemodynamic characteristics between the 2 surgical techniques. Arterial stiffness was assessed using a SphygmoCor XCEL system (AtCor Medical, Sydney, Australia), with data acquisition and analysis performed using the manufacturer’s software in the Department of Cardiovascular Investigations. Given the exploratory nature of the substudy, no propensity score matching was performed.

The following parameters were recorded: pulse wave velocity (PWV), aortic augmentation index (AIx), reflection magnitude (RM), aortic compliance, and Buckberg index. This device was selected for its validated accuracy and reproducibility in cardiovascular research. In particular, carotid–femoral PWV is considered the gold standard for noninvasive assessment of aortic stiffness and is endorsed by international clinical practice guidelines.^17–21)^ Its use in this study aimed to provide objective and comparable hemodynamic data between the surgical techniques.

### Statistical analysis

Statistical analyses were performed using GraphPad Prism v6.0.1 (GraphPad Software, San Diego, CA, USA). Quantitative variables were expressed as mean ± standard deviation or median [interquartile range], depending on normality (assessed using the Kolmogorov–Smirnov test). Categorical variables were expressed as frequencies and percentages.

Comparisons between the MWT and STR groups were made using an unpaired Student’s t-test or Mann–Whitney U test, as appropriate. Chi-squared or Fisher’s exact test was used for categorical data.

Graphs display individual patient values with medians for each group.

A multivariable logistic regression model was used to assess the association between surgical technique (STR vs. MWT) and early postoperative complications. Early postoperative complications were defined as a composite endpoint including reoperation for bleeding, stroke, prolonged intubation (>24 h), need for extracorporeal membrane oxygenation (ECMO), and in-hospital mortality. The model was adjusted a priori for age, EuroSCORE II, and preoperative aortic diameter; results are reported as odds ratios (ORs) with 95% confidence intervals (CIs).

In addition, a sensitivity analysis was performed excluding patients with an aneurysm diameter >60 mm in the STR group, to assess whether differences in outcomes were driven by extreme anatomical presentations.

## Results

### Patient characteristics

Baseline demographic, clinical, and anatomical characteristics are summarized in **Table 1** and were broadly comparable between groups. A total of 205 patients were included: 114 in the STR group and 91 in the MWT group. Mean age was similar between groups (63.7 ± 1.3 vs. 62.9 ± 1.5 years; p = 0.70). No significant differences were observed in sex distribution, body mass index (BMI), cardiovascular risk factors, or EuroSCORE II. There was no statistically significant difference in the proportion of bicuspid valves between the 2 groups (20 vs. 16; p = 0.98).

**Table 1:** Baseline characteristics

	Wrapping (n = 91)	Supracoronary tube (n = 114)	p-Value
Clinical			
Age (years)	62.9 ± 1.5	63.7 ± 1.3	0.70
Sex (male), n (%)	64 (70.3)	67 (58.7)	0.10
BMI (kg/m^2^)	29.2 ± 0.7	28.2 ± 0.67	0.30
Tobacco, n (%)	27 (29.7)	29 (25.4)	0.75
Diabetes, n (%)	16 (17.6)	12 (10.5)	0.23
Hypertension, n (%)	57 (62.6)	63 (55.2)	0.32
Dyslipidemia, n (%)	28 (30.8)	27 (23.6)	0.52
Coronary artery disease, n (%)	11 (12.2)	16 (14.0)	0.68
COPD, n (%)	11 (12.2)	8 (7.0)	0.33
EuroSCORE II (%)	1.59 ± 0.07	1.7 ± 0.07	0.29
Echocardiographic and biological			
LVEF (%)	60 ± 0.70	60 ± 0.80	0.73
Aneurysm diameter (mm)	51.8 ± 0.47	53.9 ± 0.61	**0.01**
STJ (mm)	42.3 ± 0.50	43.2 ± 0.61	0.18
Distal aorta (mm)	40.5 ± 0.47	40.8 ± 0.44	0.58
GFR by MDRD (mL/min)	83 ± 2.85	91.1 ± 2.9	**0.05**
CKD stage >1, n (%)	9 (10)	12 (10.5)	0.98
Operative			
Bicuspid aortic valve (%)	16 (17.6)	20 (17.5)	0.98
CPB time (min)	50.1 ± 3	85.7 ± 3.7	**<0.0001**
Aortic cross-clamp time (min)	28.2 ± 2	58 ± 3	**<0.0001**

The bold values indicate statistical significance (p <0.05).

BMI, body mass index; COPD, chronic obstructive pulmonary disease; LVEF, left ventricular ejection fraction; STJ, sinotubular junction; GFR, glomerular filtration rate; MDRD, modification of diet in renal disease; CKD, chronic kidney disease; CPB, cardiopulmonary bypass

### Procedural data

The mean aneurysm diameter was significantly smaller in the MWT group (51.8 ± 0.47 mm) compared to the STR group (53.9 ± 0.61 mm; p = 0.01). No aneurysms larger than 60 mm were treated using the MWT, in accordance with previous recommendations.^6–8)^ Additional anatomical parameters were comparable between groups. The sinotubular junction diameter was 42.3 ± 0.50 mm in the MWT group and 43.2 ± 0.61 mm in the STR group (p = 0.18). The distal ascending aortic diameter at the level of aortic cross-clamping was also similar between groups (40.5 ± 0.47 vs. 40.8 ± 0.44 mm; p = 0.58).

CPB and aortic cross-clamp times were significantly shorter in the MWT group (50.1 ± 3 and 28.2 ± 2 min, respectively) than in the STR group (85.7 ± 3.7 and 58.0 ± 3 min; both p <0.0001) (**Table 1**).

Intraoperative blood loss was similar between groups (MWT: 398 ± 18 mL vs. STR: 432 ± 20 mL, p = 0.21).

### Early postoperative outcomes

In-hospital mortality was 0% in the MWT group and 4.4% (n = 5) in the STR group (p = 0.06). Deaths in the STR group were related to early hemorrhagic complications (n = 2), septic shock (n = 1), early cerebrovascular accident (n = 1), and multiorgan failure following a prolonged procedure complicated by intraoperative bleeding (n = 1).

Hospital length of stay was significantly shorter in the MWT group (10.5 ± 0.3 vs. 13.5 ± 0.8 days; p = 0.001). The rate of reoperation for bleeding was higher in the STR group, although this difference did not reach statistical significance (3.3% in MWT vs. 8.8% in STR; p = 0.15). No significant differences were found in early postoperative rates of infection, prolonged intubation, intensive care unit (ICU) stay, or stroke (**Table 2**).

**Table 2:** Early postoperative outcomes

	Wrapping (n = 91)	Supracoronary tube (n = 114)	p-Value
Reoperation for bleeding, n (%)	3 (3.3)	10 (8.8)	0.15
Troponin levels H24	185.7 ± 13.8	223.5 ± 18.4	0.11
Prolonged intubation (>24 h)	2 (2.2)	8 (7.0)	0.19
Extended inotropic support (>24 h)	0 (0)	4 (3.5)	0.13
ICU stay (days)	1.71 ± 0.28	2.54 ± 0.54	0.19
ECMO support n (%)	0 (0)	4 (3.5)	0.13
Infection, n (%)	13 (14.5)	20 (17.5)	0.57
Stroke, n (%)	2 (2.2)	7 (6.1)	0.30
In-hospital stay (days)	10.5 ± 0.3	13.5 ± 0.8	**0.001**
Death at 30 days	0 (0)	5 (4.4)	0.06

The bold values indicate statistical significance (p <0.05).

H24, 24 hours; ICU, intensive care unit; ECMO, extracorporeal membrane oxygenation

ECMO support was required in 4 patients (3.5%) in the STR group and in none in the MWT group. Indications were heterogeneous and included difficulty weaning from CPB, cardiac arrest, and refractory mixed shock. Notably, 3 of these 4 patients died within 30 days.

In a subanalysis excluding the 9 patients with aneurysms >60 mm in the STR group, the mean aneurysm diameter was then comparable between groups (52.2 ± 0.58 mm in the STR vs 51.8 ± 0.47 mm in the MWT; p = 0.72). The difference in in-hospital length of stay remained highly significant (p <0.001), the 30-day mortality remained comparable (0% in MWT vs. 3.8% in STR; p = 0.12), the reoperation rate for bleeding remained nonsignificant (3.3% in MWT vs. 5.7% in STR; p = 0.51), and no significant difference was observed in infection rates.

### Outcomes multivariable analysis

In multivariable logistic regression analysis, surgical technique was not independently associated with early postoperative complications after adjustment for age, EuroSCORE II, and preoperative aortic diameter (STR vs. MWT: OR 2.10, 95% CI 0.80–5.51; p = 0.126). However, a nonsignificant trend toward a higher rate of early complications was observed in the STR group (**Table 3**).

**Table 3:** Multivariable logistic regression analysis for early postoperative complications

Variable	OR	95% Confidence Interval	p-Value
Surgical technique (STR vs. MWT)	2.10	0.80–5.51	0.126
Aortic diameter (per mm)	1.02	0.95–1.09	0.626
EuroSCORE II (per unit)	1.00	0.92–1.08	0.948
Age (per year)	0.99	0.96–1.03	0.765

Early postoperative complications were defined as a composite of reoperation for bleeding, stroke, prolonged intubation, ECMO requirement, and in-hospital mortality.

OR, odds ratio; STR, supracoronary tube replacement; MWT, modified wrapping group; ECMO, extracorporeal membrane oxygenation

### Outcomes during follow-up

**Fig. 2:**
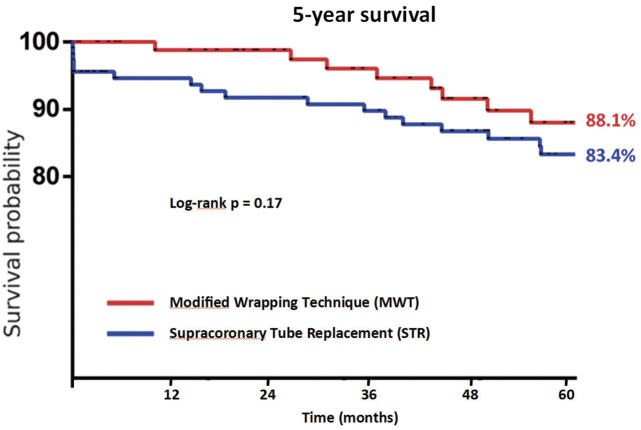
Kaplan–Meier curves for all-cause mortality according to the 2 surgical techniques. The 5-year survival was similar between the 2 groups (88.1% in MWT vs. 83.4% in STR, p = 0.17).

At 5 years, survival was 88.1% in the MWT group and 83.4% in the STR group. Kaplan–Meier analysis showed no statistically significant difference (p = 0.17, log-rank test). Survival curves diverged slightly early postoperatively but remained parallel thereafter (**Fig. 2**).

During follow-up between 30 days and 5 years, the incidence of endocarditis or prosthetic infection was significantly lower in the MWT group (1.1% vs 7.0%; p = 0.04). In this retrospective dataset, endocarditis and prosthetic infection could not be reliably distinguished and were therefore analyzed as a single composite endpoint. One patient in the MWT group required reoperation for prosthetic infection, compared with 4 patients in the STR group. Among these 4 patients, 2 cases of postoperative mediastinitis were observed and, after multidisciplinary evaluation, were classified as prosthetic infections due to the proximity to the prosthetic material; both patients died during hospitalization.

No significant differences were observed in the rates of acute dissection, reoperation for dilatation, or aneurysm recurrence (**Table 4**). In the MWT group, no redilatation of the treated AA was observed. One case of aortic arch dilatation was identified and required reoperation. In the STR group, 4 cases of redilatation requiring reoperation were observed. These dilatations did not reach the surgically treated portion of the AA but were located in the distal AA—a segment not replaced due to constraints related to aortic cross-clamping and the arterial cannulation site—and, in 3 cases, extended to the aortic arch.

**Table 4:** Efficacy outcomes during follow-up

	Wrapping (n = 91)	Supracoronary tube (n = 114)	p-Value
Acute dissection, n (%)	1 (1.1)	3 (2.6)	0.63
Reoperation for dilatation in the treated aortic segment, n (%)	0 (0)	4 (3.5)	0.13
Endocarditis or prosthesis infection, n (%)	1 (1.1)	8 (7.0)	**0.04**
Median variation of AA diameter (in mm)	−19.4 ± 0.92	−18.35 ± 0.9	0.55

The bold values indicate statistical significance (p <0.05).

AA, ascending aorta

The 5-year CT scan showed no significant aortic wall thinning in the MWT group.

### Hemodynamic outcomes (subgroup of 40 patients, 20 per group)

The following parameters were recorded:

- PWV: Carotid–femoral PWV was calculated as distance divided by transit time; higher values reflect greater stiffness.
- AIx: The ratio of augmentation pressure to pulse pressure (PP), expressed as a percentage; higher values reflect greater stiffness.
- RM: The proportion of the forward pressure wave reflected back toward the heart; higher values reflect greater stiffness.
- Aortic compliance: The ability of the aorta to expand and contract in response to blood flow, calculated as stroke volume (SV) divided by PP (SV/PP); lower values indicate increased stiffness.
- Buckberg Index: The ratio of diastolic pressure–time integral (DPTI) to systolic pressure–time integral (SPTI); values <0.8 suggest subendocardial ischemia.

**Fig. 3:**
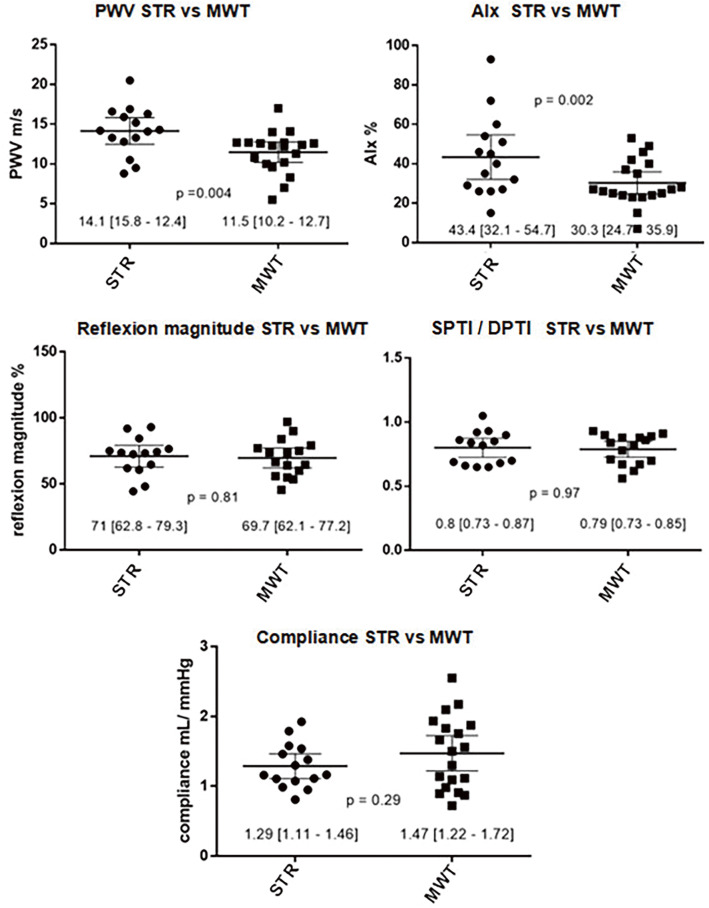
Comparison of postoperative neo-aorta dynamics according to the surgical technique. Both PWV and AIx were significantly lower in the MWT group compared to the STR group (11.5 ± 2.7 vs. 14.1 ± 3 m/s, p = 0.004 and 30.3% ± 11.7% vs. 43.4 ± 20.4%, p = 0.002). The reflection magnitude (p = 0.81), the aortic compliance (p = 0.42), and the Buckberg index (p = 0.19) were similar between groups. PWV, pulse wave velocity; AIx, aortic augmentation index; MWT, modified wrapping technique; STR, supracoronary tube replacement; DPTI, diastolic pressure–time integral; SPTI, systolic pressure–time integral

PWV was significantly lower in the MWT group (11.5 ± 2.7 m/s) compared to the STR group (14.1 ± 3.0 m/s; p = 0.004). The AIx was also reduced in the MWT group (30.3% ± 11.7% vs. 43.4% ± 20.4%; p = 0.002). No significant differences were observed in RM, aortic compliance, or the Buckberg index (**Fig. 3**).

## Discussion

In this retrospective cohort of patients with isolated ascending aortic aneurysms, the present study compared the clinical outcomes of MWT and STR in a real-world surgical setting. Despite the absence of randomization, 5-year survival was similar between the 2 techniques, with no statistically significant difference observed. MWT was associated with the absence of in-hospital mortality and shorter operative times. Overall, the 5-year results suggest potential advantages of the modified wrapping approach, including lower rates of prosthetic infection and favorable arterial dynamic parameters, in a selected patient population.

### Study population considerations

To eliminate confounding from concomitant aortic valve interventions, this study included only patients undergoing isolated ascending aortic surgery. This design was motivated by prior reports showing that aortic valve replacement improves blood flow patterns and reduces aortic wall stress^22)^ and arterial stiffness.^23–25)^ Both surgical techniques employed the same prosthetic material to allow comparability and isolate the effect of the surgical approach. To our knowledge, this study represents one of the few large-scale comparisons of these 2 surgical techniques performed without concomitant aortic valve procedures, thereby allowing for a more isolated evaluation of outcomes related to AA repair strategies.

Notably, the mean aneurysm diameter in both groups was lower than the 55-mm threshold recommended in the clinical practice guidelines in force during the study period.^2,3)^ However, this aligns with emerging evidence supporting earlier intervention to prevent acute aortic events.^26,27)^ Importantly, the surgical indications in this study reflect the clinical practices during the inclusion period and are consistent with recently updated European and international guidelines, which increasingly support earlier intervention in selected patients.^28)^

It should be acknowledged that the mean ascending aortic diameter was smaller in the MWT group than in the STR group (51.8 ± 0.47 vs. 53.9 ± 0.58 mm; p = 0.01), reflecting contemporary clinical practice in which wrapping is generally reserved for moderately dilated aneurysms, in accordance with previously reported indications.^6–8)^ This predefined anatomical selection should be considered when interpreting the comparative outcomes between techniques.

To further address potential selection effects related to aneurysm size, an additional analysis was performed in the STR subgroup after exclusion of aneurysms larger than 60 mm. In this anatomically restricted comparison, the difference in length of hospital stay between techniques remained significant, whereas early mortality, reoperation for bleeding, and infection rates were comparable. These findings support that the observed differences in perioperative efficiency are not solely attributable to baseline differences in aneurysm size.

Importantly, additional anatomical parameters, including sinotubular junction and distal ascending aortic diameters, were comparable between groups, reinforcing the anatomical consistency of the comparison beyond maximal diameter.

### Safety outcomes

Regarding perioperative safety, early outcomes were comparable between the 2 surgical strategies. No in-hospital mortality was observed in the MWT group. Major postoperative complications occurred at similarly low rates, and the use of the MWT was not associated with an increased risk of early adverse events in this selected patient population.

These findings are consistent with previously published series reporting reoperation rates for bleeding ranging from 1.5% to 6.2% after wrapping procedures.^7,8,15,29)^ In the STR group, the observed rate of reoperation for bleeding is consistent with rates reported in the literature.^7,30,31)^ However, it appears higher than that described in some contemporary series, such as those reported by Idrees et al. (4.1%)^32)^ and Kim et al. (1.4%).^15)^ Even after exclusion of patients with aneurysms larger than 60 mm, the reoperation rate for bleeding in the STR cohort remained at 5.7%. These inter-study differences may reflect variations in patient selection, aneurysm size, surgical complexity, and perioperative management strategies.

Similarly, 30-day mortality was 0% in the MWT group versus 4.4% in the STR group (p = 0.06), a difference that did not reach statistical significance but remains clinically noteworthy. These rates are consistent with previously reported large series in ascending aortic surgery.^3,6,30,31)^ After exclusion of patients with aneurysms larger than 60 mm, 30-day mortality in the STR group decreased to 3.8%, yet remained numerically higher than that reported in large contemporary series, such as those by Kim (0%)^15)^ and Idrees (0.51%).^32)^ While improvements in surgical techniques and perioperative care over time may partly account for these differences, aneurysm size appears to be a key explanatory factor. In both the Kim and Idrees series, the mean mid-ascending aortic diameter was substantially smaller (49.5 and 48 mm, respectively) than in our STR cohort (53.9 mm). These observations support the growing body of evidence suggesting that earlier surgical intervention in ascending aortic aneurysms may reduce early postoperative complications, including reoperation for bleeding and early mortality.^26–28)^ The higher incidence of ECMO in the STR group should be interpreted with caution, as it was based on a small number of heterogeneous and severe clinical situations, and is likely unrelated to the surgical technique itself.

Five-year survival did not differ significantly between the 2 groups, although a numerical trend favoring the MWT group was observed (88.1% vs. 83.4%; log-rank p = 0.17). Importantly, this difference was driven primarily by events occurring in the early postoperative period, as survival curves diverged minimally thereafter. Similar patterns have been reported in previous studies,^7,33)^ underscoring the need for larger, adequately powered investigations to confirm these findings.

### Efficacy outcomes

Only the rate of endocarditis or prosthetic infection reached statistical significance, with 1.1% in the MWT group versus 7.0% in the STR group (p = 0.04). While data on postoperative infections after AA surgery are limited, our results are broadly consistent with the ranges reported in the literature—3.8%–5% for STR^30,31,34,35)^ and 0%–2% for wrapping.^6,7,24)^ This difference may be explained by the extravascular position of the prosthesis in the MWT technique, which limits direct contact with circulating blood.

In contrast to previous wrapping studies, particularly the findings reported by Kim et al.^15)^ in their large cohort study comparing 96 patients treated with wrapping and 108 with ascending aortic replacement, we did not observe any trend toward redilatation of the proximal aortic arch following MWT. This discrepancy may be explained, at least in part, by the fact that in Kim’s series both wrapping and tube replacement were systematically combined with aortic valve replacement, which may have potentially influenced postoperative remodeling. Alternatively, this difference could be attributed to the specific features of the MWT used in our study. By ensuring comprehensive coverage of the suture lines and the entire AA, and by performing the procedure while maintaining the aorta under physiological pressure, our modified wrapping approach may reduce the risk of secondary dilatation and potentially provide more homogeneous reinforcement of the aortic wall. In the STR group, the persistence of an untreated distal ascending aortic segment—due to technical constraints related to cross-clamping and arterial cannulation—may have contributed to secondary progression in this area. However, no statistically significant difference was observed between groups, and reinterventions appeared to be primarily associated with disease progression in untreated segments, particularly the aortic arch.

Concerns have been raised in the literature about prosthetic migration^12)^ and plication^13,14)^ following wrapping. However, neither phenomenon was observed in our cohort of MWT during the follow-up. The absence of migration may be attributed to the strong proximal anchoring of the prosthesis in our modified procedure, and the lack of plication likely results from careful tensioning and intraoperative verification during placement. Consistently, no cases of aortic wall thinning were observed in this cohort after wrapping.

### Aortic dynamics

Hemodynamic assessments demonstrated favorable arterial parameters in the MWT group. Among the various stiffness indices, PWV is the most validated and reproducible,^17–19,24)^ and was therefore used as the primary outcome measure. Both PWV and the AIx were significantly lower in the MWT group (p = 0.004 and 0.002, respectively). However, the small number of patients in each group limits the statistical power of these findings, and larger studies are needed to confirm the observed hemodynamic differences.

These differences may be attributed to the biomechanical properties of the surgical repair. The rigid prosthesis used in STR replaces the native aorta and lacks viscoelasticity, which may impair ventriculo-arterial coupling. In contrast, the wrapping technique preserves the native aortic wall and its viscoelastic behavior, potentially improving long-term vascular compliance.

Despite these limitations, our findings—together with prior reports—support the safety and efficacy of the MWT. This approach may represent a viable alternative in selected patients, particularly those with comorbidities or smaller aneurysms (<60 mm), offering comparable clinical outcomes while potentially preserving vascular function.

### Limitations

This study has several limitations that should be acknowledged.

First, the retrospective design and the absence of randomization inherently expose the comparison between MWT and STR to selection bias. These results reflect outcomes from a high-volume center with specific expertise in MWT, which may limit generalizability. The decision to perform MWT or STR was left to the operating surgeon based on clinical judgment, aortic diameter, patient age, and operative risk. This may have resulted in unmeasured confounding despite comparable baseline characteristics. Selection bias related to surgical decision-making cannot be fully excluded. However, the consistency of outcomes across adjusted analyses and predefined subgroups provides partial reassurance regarding the internal validity of the comparison. No dedicated subgroup analyses were performed for patients with bicuspid aortic valve, and the study was not powered to assess outcomes specifically within this population.

Furthermore, endocarditis and prosthetic infection could not be consistently distinguished in all cases and were therefore combined into a single endpoint, which may have limited the granularity of infection-related outcome analyses.

Second, the sample size, although reasonable for a surgical study, may be insufficient to detect differences in rare adverse events. For example, although trends in mortality, reoperation, and infection rates were observed, some did not reach statistical significance. Larger, multicenter cohorts would enhance statistical power and generalizability.

Third, the hemodynamic substudy was conducted on a relatively small group of 40 patients, with only 20 patients in each arm, and did not rely on propensity score matching. As a result, despite efforts to select patients with comparable baseline characteristics, residual confounding cannot be excluded. While this substudy offers meaningful insights into arterial stiffness and ventriculo-arterial coupling, its findings should be interpreted with caution. Consequently, these hemodynamic results should be regarded as exploratory and hypothesis-generating; their potential clinical impact, particularly in terms of left ventricular remodeling and heart failure outcomes, remains to be established and warrants confirmation in larger, prospectively designed studies.

In addition, the SphygmoCor system was not available throughout the study period, which may have introduced temporal bias, as only the most recent patients were included.

Fourth, although 5-year survival was analyzed, imaging data at equivalent time points were not available for all patients. Systematic assessments at predefined intervals would allow a more robust evaluation of anatomical outcomes, including the occurrence of redilatation or migration.

Finally, the study did not assess functional status or quality of life postoperatively, which are increasingly recognized as key outcomes in aortic surgery. Future investigations should include validated clinical endpoints beyond morbidity and mortality.

Accordingly, this study should not be interpreted as a definitive comparative effectiveness analysis, but rather as an assessment of the safety and clinical performance at 5 years of MWT compared with STR in a selected real-world population.

## Conclusion

In this 5-year comparative retrospective analysis of isolated ascending aortic aneurysms, the MWT appears to offer outcomes comparable to STR, with the added benefits of shorter operative times, lower prosthesis-related infection rates, and improved aortic stiffness parameters. Five-year survival was similar between the 2 techniques, with no statistically significant difference observed. These findings support the MWT not as a substitute for STR, but as a complementary surgical option in carefully selected patients with moderately dilated AAs. Despite the study’s limitations, the observed differences in arterial stiffness parameters between techniques provide preliminary insights into the potential biomechanical implications of ascending aortic repair strategies. However, larger multicenter prospective studies are needed to confirm these results, evaluate long-term durability, and better define the role of MWT in contemporary aortic surgery.

## Declarations

### Data availability

The data that support the findings of this study are available from the corresponding author upon reasonable request.
